# Supplementary material for: CircXPO1 Promotes Glioblastoma Malignancy by Sponging miR-7-5p
Source: Cells. 2023 Mar 8;12(6):831. doi: 10.3390/cells12060831 (PMC10047377; doi:10.3390/cells12060831)
Supplement: Supplementary file 1 [file cells-12-00831-s001.zip › cells-2126998-supplementary.pdf]

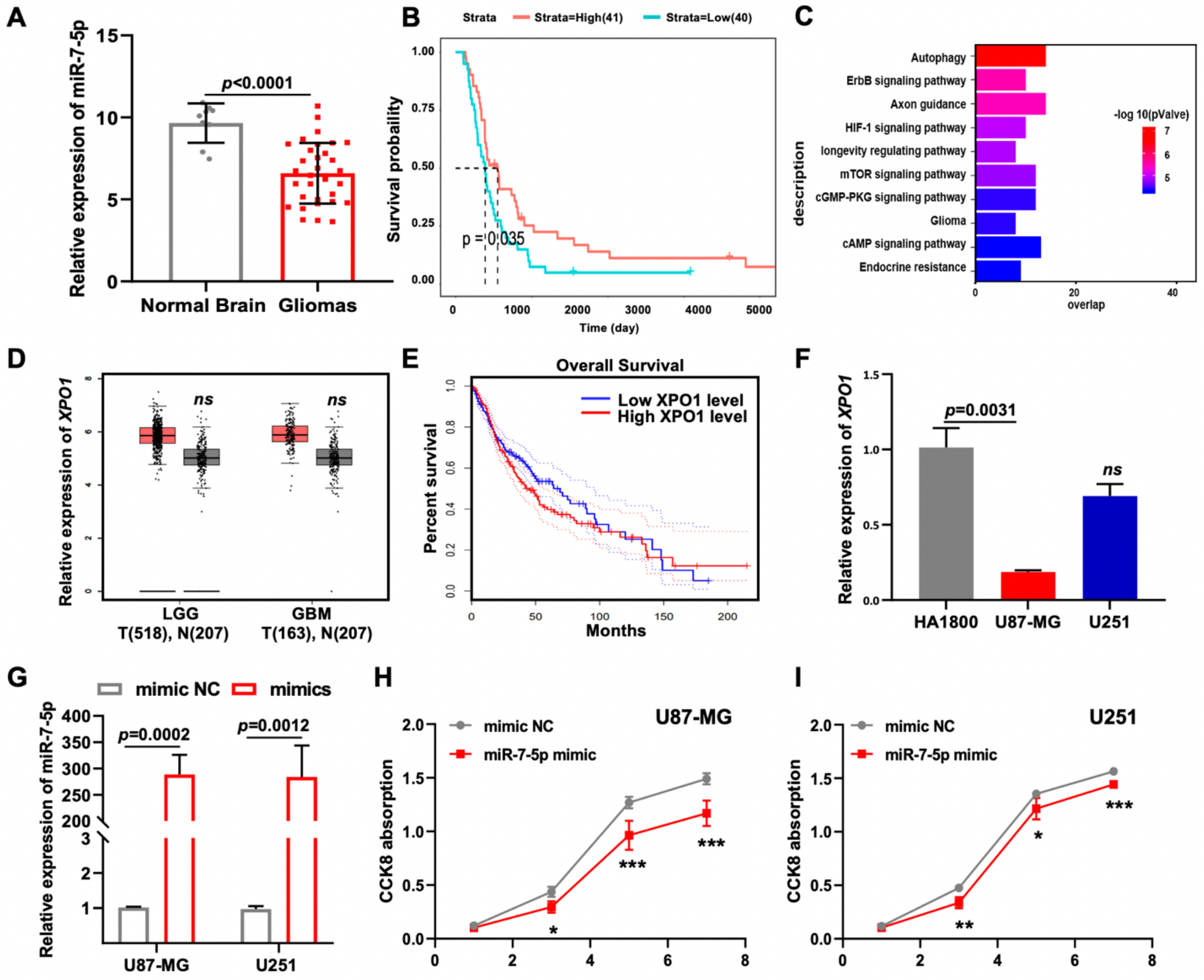

Figure S1. Analysis of XPO1 expression in glioblastoma and growth inhibition of miR-7-5p mimics in glioblastoma cells. A. Decreased expression of miR-7-5p in glioblastoma tissues was demonstrated with CGGA data. **B.** Kaplan–Meier survival analysis validated high levels of miR-7-5p in glioma patients were associated with good prognosis. **C.** Pathway analysis investigated downstream genes and potential mechanisms of miR-7-5p. **D.** The expressions of XPO1 were not significantly increased in LGG and GBM tissues vs. normal controls. **E.** Kaplan-Meier survival analysis indicated that high XPO1 level was not positively correlated with shorter overall survival time in glioblastoma patients. **F.** The expressions of XPO1 were not significantly increased in glioblastoma cell line U87-MG and U251. **G.** Transfection of miR-7-5p mimics raised the microRNA levels in glioblastoma cells. **H, I.** Growth curves of glioblastoma cells with or without miR-7-5p mimics were plotted by CCK-8 assays. "*"indicates $p < 0.05$; "**" indicates $p < 0.01$; "***" indicates $p < 0.001$.

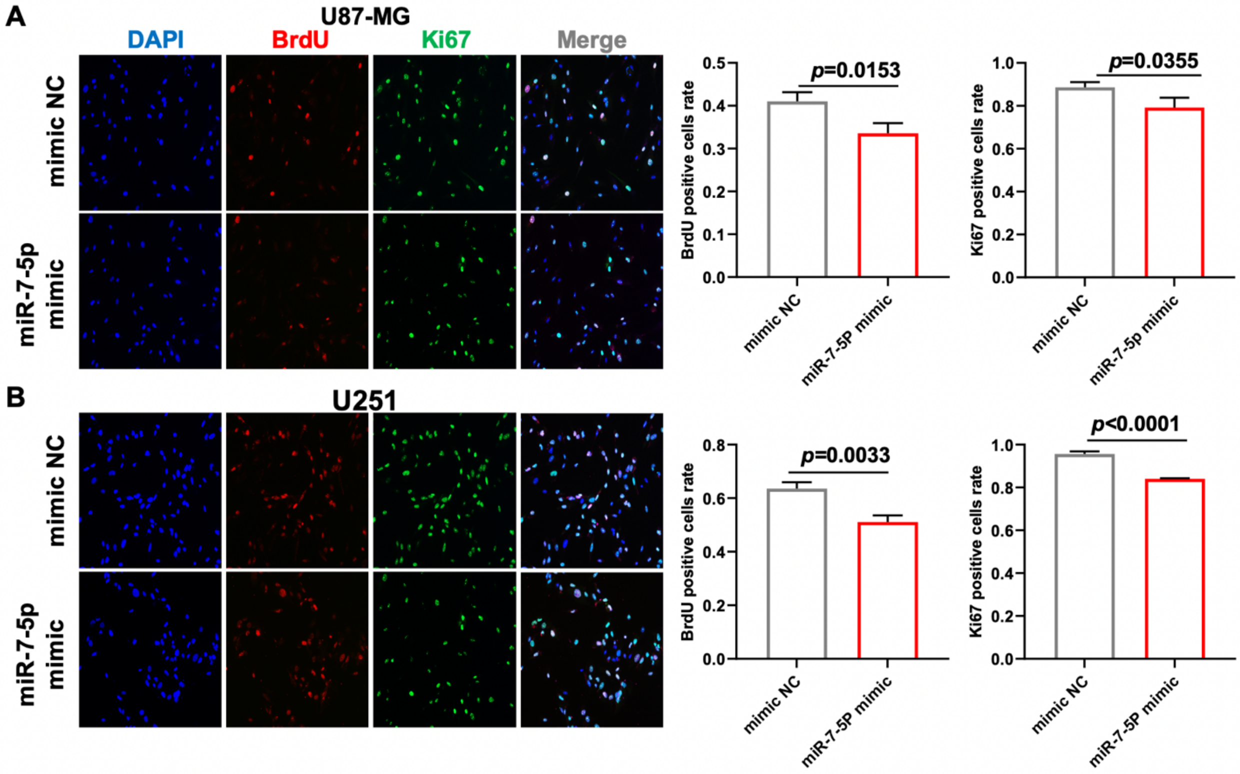

Figure S2. Immunofluorescent staining showed the Ki67 and BrdU positive cells in miR-7-5p mimics treated U87-MG **(A)** and U251 **(B)** cells. The rates of BrdU and Ki67 positive cells were calculated with ImageJ on the right side.

Table S1. CircXPO1 shRNA sequences.

Primer	Sequence(5'-3')
hsa-circ-XPO1-sh1	GTGCGAAGTAATCTATGCCAGC
hsa-circ-XPO1-sh2	CCAAGGAACCAGTGCGAAGTAATC
scramble	GGGCGACTTAACCTTAGG

Table S2. Primer sequences for the study.

Primer	Sequence(5'-3')
CircXPO1-F1-155	CAGTGCGAAGTAATCTATGCCA
CircXPO1-R1-155	GAGCCATTCTTTGCTGGGCT
XPO1-F2-246	AGTCGAATGGCTAAACCAGAGG
XPO1-R2-246	GGAGCCTATTGCCCAACACA
gXPO1-R3-189	TCCTAAAATGTATTTCCACCCCAGA
GAPDH-F-212	CCAAGGAGTAAGACCCCTGG
GAPDH-R-212	TGGTTGAGCACAGGGTACTT
hsa-miR-7-5p-F	GCCGTGGAAGACTAGTGATTT
hsa-miR-23a-3p-F	CCACTATCACATTGCCAGGGA
hsa-miR-106b-3p-F	CCGCACTGTGGGTACTTGC
hsa-miR-551b-5p-F	GAAATCAAGCGTGGGTGAGAC
hsa-miR-1248-F	CGCACCTTCTTGTATAAGCAC
U6-F	TGCTCGCTTCGGCAGCACAT
U6-R	ACGCTTCACGAATTTGCGTGTC
RB1-F-214	CTCTCGTCAGGCTTGAGTTTG
RB1-R-214	GACATCTCATCTAGGTCAACTGC
AKT3-F-130	AATGGACAGAAGCTATCCAGGC
AKT3-R-130	TGATGGGTTGTAGAGGCATCC
Raf1-F-165	GGGAGCTTGGAAGACGATCAG
Raf1-R-165	ACACGGATAGTGTTGCTTGTC
KLF4-F-136	ACCTACACAAAGAGTTCCCATC
KLF4-R-136	TGTGTTTACGGTAGTGCCTG
SP1-F-118	GTGGAGGCAACATCATTGCTG
SP1-R-118	GCCACTGGTACATTGGTCACAT
